# Spanish Translation and Dissemination of EMPOWER Materials to Address Barriers to Pain Management at the End of Life

**DOI:** 10.1089/pmr.2023.0090

**Published:** 2024-04-15

**Authors:** John G. Cagle, Iraida Carrion, Todd D. Becker, Peiyuan Zhang

**Affiliations:** ^1^ University of Maryland School of Social Work, Baltimore, Maryland, USA.; ^2^ University of South Florida School of Social Work, Tampa, Florida, USA.

**Keywords:** disparities, end-of-life care, Hispanic patients, hospice, opioids

## Abstract

**Introduction:**

The Effective Management of Pain by Overcoming Worries to Enable Relief (EMPOWER) intervention is an evidence-supported approach for addressing barriers to pain management (e.g., patient/family concerns about addiction) at the end of life. Such barriers appear more pronounced among Spanish-speaking individuals. This study aimed to (1) translate EMPOWER materials into Spanish, (2) disseminate materials to hospices with ≥25% Hispanic patients, and (3) survey hospices about the use and usefulness of materials.

**Methods:**

We back translated EMPOWER materials with harmonization, then disseminated materials to 242 hospices. Thereafter, we used a semistructured survey to assess use and usefulness of EMPOWER materials using univariate statistics and content analysis.

**Results:**

Thirty-eight hospice representatives responded (participation rate = 15.7%). Respondents were primarily non-White (55.3%) and Hispanic (60.5%). Nealy half (47.4%) were nurses. A majority (81.6%) indicated they currently employ ≥1 full-time English–Spanish bilingual team member. Among those who reported receiving the EMPOWER materials (*n* = 29), 58.6% indicated they—or another staff member—used them with patients or families. Using a single-item rating (0 = *not useful* to 10 = *very useful*), respondents evaluated the English EMPOWER materials' usefulness as 7.6 (standard deviation [SD] = 1.4) and Spanish materials as 8.4 (SD = 1.4). Most (62.1%) indicated they would likely use EMPOWER materials in the future.

**Conclusion:**

Thematic findings suggest EMPOWER reinforces clinical education, promotes discussion about pain management, and helps address culturally specific barriers to care. EMPOWER appears to be a useful, easy to use, and promising intervention that can be implemented among both English- and Spanish-speaking populations.

## Background

Within the United States, hospice agencies provide care to an estimated 1.7 million dying patients annually. The effective treatment of pain is a high priority for hospice providers and a common goal for patient-centered care. Unfortunately, challenges to pain management persist in hospice. Common obstacles include poor communication, a lack of education, and misperceptions among patients and/or their informal caregivers.^2–4^ Patients, for example, may avoid taking effective pain medicines because they worry about addiction, overdose, or being perceived as weak for complaining about pain. Prompting conversations among patients, family caregivers, and hospice professionals about these barriers can help overcome them.

The Effective Management of Pain by Overcoming Worries to Enable Relief (EMPOWER) approach is a promising and efficacious educational intervention that addresses these common barriers and improves outcomes for both patients and caregivers.^5,6^ Results from a randomized clinical trial suggest that the EMPOWER approach can improve caregiver knowledge about pain management, lessen concerns about pain and pain medications, and reduce patient pain.^5^

Evidence also suggests EMPOWER may be especially beneficial for underrepresented groups, such as African Americans, by reducing stigma related to requesting pain treatment (e.g., concerns about being perceived as “drug-seeking”).^5^ The core elements of EMPOWER are: (1) a screening form identifying patient/caregiver barriers; and, (2) an educational brochure including evidence-based information to address any barriers identified during the screen.

Unfortunately, before the study herein, EMPOWER materials were available only in English. This is problematic because according to the U.S. census, there are 63.5 million native Spanish speakers in the United States.^7^ In addition, an estimated 100,000 hospice patients annually are of Hispanic descent,^8^ and most households are thought to have at least one native Spanish speaker. Research further demonstrates that Spanish-speaking individuals may be especially susceptible to pain management barriers.

For instance, a national survey found a greater proportion of Hispanic respondents (vs. non-Hispanic respondents) believed: pain medicine cannot really control pain (73% vs. 24%), pain is a sign of weakness (56% vs. 10%), and good patients do not talk about their pain (46% vs. 18%).^9^ These misconceptions are addressed in the EMPOWER brochure. Furthermore, Hispanic patients are more likely to experience severe pain^10^ and less likely to receive adequate treatment.^11^

Taken together, the existing evidence suggests that the EMPOWER approach has strong potential to overcome address barriers to pain management within the Hispanic population, provided the intervention materials are translated appropriately with careful attention to social and linguistic contexts. To address this need, the specific aims of this study were to:

Aim 1. Translate the EMPOWER materials into Spanish using a validated back-translation approach with harmonization.
Aim 2. Disseminate EMPOWER materials (both English and Spanish) by mailing printed samples to hospices with ≥25% Hispanic patients; and collect preliminary evidence on the perceived usefulness and feasibility of the EMPOWER approach using follow-up surveys—through phone or email—with these hospices.

In addition, the survey also provided data on availability of bilingual (English–Spanish) clinical staff, an understudied topic in the hospice field.

## Methods

### English-to-Spanish translation

The EMPOWER materials underwent back-translation with subsequent harmonization,^12^ which has been successfully used by the study team in prior translation efforts.^9,13^ Using this method, coauthor I.C., a native English and Spanish speaker (Puerto Rican) and seasoned hospice researcher with extensive clinical health care experience, initially translated EMPOWER materials from English into Spanish. Then, a group of five English–Spanish bilingual individuals representing linguistically diverse regions (i.e., Colombia, Honduras, Mexico/Peru, and Venezuela) reviewed the initial draft before back translating the materials into English.

The research team then compared the back-translated materials with one another, as well as with the original source material to identify inconsistencies. Specifically, the research team sought terms or passages requiring harmonization or reconciliation between the back translations and source material.^12^ Translators were compensated $200.

After identifying translated excerpts requiring harmonization, the research team coordinated with back translators to reconcile unclear terms/passages, and inconsistencies were discussed until reaching consensus. After a tie, the initial translator (coauthor I.C.) made the final decision about Spanish wording.

### Dissemination to and survey of hospices

#### Sampling approach

Our team used publicly available data from the most recently published 2018 Hospice Provider of Services file^8^ to compile a list of U.S. hospices reporting an annual patient census of ≥25% Hispanic. The file includes available addresses and phone numbers for 100% of Medicare-certified hospices.

#### Procedures

Our team color-printed trifold EMPOWER brochures (English and Spanish) using cardstock, which included large-print text and professional graphic design. The EMPOWER screening form was printed on 8.5 × 11” white paper. Using contact information from the agency list, project managers (coauthors T.D.B. and P.Z.) contacted hospice providers by phone two weeks before the planned dissemination of materials to identify a point of contact (POC) to receive the mailed materials. During this call, our team confirmed the agency mailing address and solicited an email address from the POC to provide them with PDF proofs of EMPOWER materials.

Materials were tailored with the unique name and contact information for a given hospice. In case of POC unavailability, materials were mailed to the “Hospice Director or Director of Nursing” using a confirmed valid address. EMPOWER mailings were sent to all viable hospices listed in the sampling frame. Mailings included an introductory letter, a figure showing basic EMPOWER procedures, 10 Spanish brochures, 10 English brochures, 10 Spanish screens, 10 English screens, and the initial EMPOWER study.^5^

#### Measures

The usefulness and feasibility of the disseminated EMPOWER materials were assessed through a follow-up survey with hospice representatives approximately three to four months after the initial mailing. Material mailouts to hospice agencies began in Fall 2021 and follow-up surveys, administered by phone or email, concluded Winter 2022. Survey data were captured using Qualtrics survey software (Version October 2021).^14^ Survey respondents were asked about perceptions of the EMPOWER materials, as well as Spanish language resources for patients and the availability of bilingual (English–Spanish) clinical staff at their agency.

The full survey measure and item list are provided in Supplementary Appendix S1. Authors J.G.C., T.D.B., and P.Z. conducted follow-up interviews and facilitated survey administration through email. All project methods and procedures were reviewed and approved by the University of Maryland, Baltimore Institutional Review Board (IRB #00092797).

#### Analysis

The number of both substantive and negligibly discrepant terms identified during the back translation with harmonization process is reported separately for the EMPOWER brochure and screen. For substantive terms and phrases, the adjudication rationale used for making final decisions during harmonization is described. The finalized versions of Spanish-translated EMPOWER materials are available by request from the corresponding author.

To assess survey results, descriptive statistics (means, standard deviations [SDs]; frequencies, percentages) appropriate to the measurement level are reported for quantitative survey data. Then, following Krippendorff's content analysis approach,^15^ open-ended responses were thematically categorized, providing an anecdotal context for findings.

## Results

### Back translation with harmonization

#### EMPOWER brochure

Back-translation procedures revealed 50 instances in which brochure content diverged from the original English text. Most (*n* = 31, 62.0%) reflected negligible differences with little effect on the excerpt's meaning (original English: “once the pain goes away” vs. back-translator English: “once the pain disappears”). Often, these instances were seen in only one or two back translations, so the original translation remained unchanged, but differences were considered substantive when back translations altered the original intended meaning (see Table 2 for selected examples).

Most discrepancies were resolved by replacing clinical terms (e.g., “analgesics”), with lay language (e.g., “medications for pain”). Other translational differences appeared throughout the brochure. For instance, our original translation of “manage comfort” was back translated as “manage well-being,” “focus on a person's well-being,” “ensure well-being,” and “manage wellness.” By extension, “discomfort” was back translated as “pain,” “bother,” and “inconvenience,” idiosyncrasies that highlighted differences between clinical and lay meanings.

As such, “manage comfort” was replaced with more concrete language (i.e., “manage pain relief”), and to reconcile discrepancies with the term “discomfort,” we used the term endorsed most frequently by back translators when seeking feedback on the prospective final version. Finally, all translators reviewed and approved the final version.

#### EMPOWER screen

Six translational discrepancies were identified during translation of the EMPOWER screen, two (33.3%) of which were considered substantive (Table 2). Item #1, which covers patient concerns about addiction, was back translated as “becoming an addict” and “becoming a drug addict.” Thus, we clarified the intended adjectival meaning by moving “to the medications” to the question item. Furthermore, item #5 that assesses concerns about “being perceived as a drug-seeker.” Although the original Spanish translation prompted back translations of “drug addict,” we opted for a more literal translation. As with the brochure, back translators approved all harmonization decisions.

### Dissemination to and survey of hospices

**FIG. 1.**
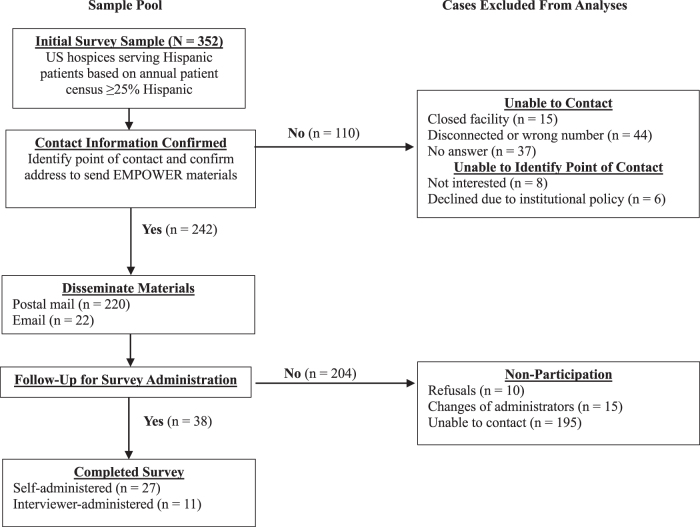
Flowchart describing the analytic study sample and case exclusions.

We identified 352 hospices whose annual patient census was ≥25% Hispanic based on the Hospice Provider of Services file,^8^ most of which were located in California (*n* = 191, 54.3%) and Texas (*n* = 127, 36.1%). Figure 1 provides a flowchart illustrating contact attempts, vetting of the sampling frame, and known reasons for noncontact/nonparticipation. Ultimately, we disseminated EMPOWER materials to 242 hospices (68.8% of agencies listed in the original sampling frame), 220 of which received mailed hardcopies, with the remaining 22 agencies receiving PDF copies of the materials through email.

In total, 38 hospice representatives completed the follow-up survey. Based on the 242 hospices that received the EMPOWER materials, the resulting survey participation rate was 15.7%. Most surveys (*n* = 27, 71.1%) were self-administered, whereas the remainder (11, 28.9%) were interviewer facilitated over the phone.

#### Sample description

As Table 1 shows, survey respondents were, on average, 42.8 years of age (SD = 9.9), largely (84.2%) female, and racially and ethnically diverse, with a majority non-White (55.3%) and Hispanic (60.5%). Most (44.7%) respondents described their job role as both clinical and administrative, relative to exclusively clinical (36.8%) or administrative (18.4%) roles. Regarding professional discipline, just under half of respondents (47.4%) were from nursing, followed by social work (21.1%), medicine (13.2%), or another professional role (18.4%). Most respondents (86.9%) indicated their agency currently employs at least one full-time clinical team member who is bilingual in English and Spanish.

**Table 1.** Participant Characteristics and Quantitative Survey Results (*N* = 38)

Variable	n (%)
Age, mean (SD), year	42.8 (9.9)
Gender	
Male	6 (15.8)
Female	32 (84.2)
Ethnicity	
Hispanic or Latino	15 (39.5)
Not Hispanic or Latino	23 (60.5)
Race	
Asian	4 (10.5)
Black or African American	3 (7.9)
White	17 (44.7)
Multiracial	4 (10.5)
Other	9 (23.7)
Missing	1 (2.6)
Job role	
Clinical	14 (36.8)
Administrative	7 (18.4)
Clinical and administrative	17 (44.7)
Professional discipline	
Physician	5 (13.2)
Nurse	18 (47.4)
Social worker	8 (21.1)
Administrator or other professional discipline	7 (18.4)
Approximately how many bilingual (English–Spanish) hospice team members are employed at your agency?	
0	5 (13.1)
1	3 (7.9)
2	5 (13.1)
3–5	12 (31.6)
≥6	13 (34.2)
Which of the following patient care disciplines have bilingual (English–Spanish) hospice speakers at your agency?^a^ [Yes]	
Medicine	19 (50.0)
Nursing	31 (81.6)
Nursing aide	19 (50.0)
Social work	21 (55.3)
Pharmacy	12 (31.6)
Chaplain	21 (55.3)
Would you say that your hospice employs enough Spanish-speaking clinical staff to meet the needs of your patients?^a^	
Yes	22 (71.0)
No	9 (29.0)
Received EMPOWER materials through mail or email	
Yes	29 (76.3)
No	9 (23.7)
Agency used EMPOWER materials with patients or families^b^	
Yes	17 (58.6)
No	9 (31.0)
Unsure	3 (10.3)
Perceived usefulness of EMPOWER materials (0 = *not useful* to 10 = *very useful*)^b^	
English, mean (SD)	7.6 (1.4)
Spanish, mean (SD)	8.4 (1.4)
Are you likely to use the EMPOWER materials in the future?^b^	
Yes	18 (62.1)
No	11 (37.9)

^a^ Items asked only of those who confirmed having at least one Spanish-speaking clinical staff member (*n* = 31).

^b^ Items asked only of those who confirmed having received EMPOWER materials (*n* = 29).

EMPOWER, Effective Management of Pain by Overcoming Worries to Enable Relief; SD, standard deviation.

**Table 2.** Selected Substantive Passages for Harmonization from the Translation and Back Translation of Effective Management of Pain by Overcoming Worries to Enable Relief Materials

Source	Original English phrasing	Initial Spanish translation	Discrepant English back-translations	Harmonized Spanish phrasing
EMPOWER brochure	Manage comfort	*Manejar el bienestar*	Manage well-being	*Manejar el alivio del dolor*
			Focus on a person's well-being	
			Ensure well-being	
			Manage wellness	
	Discomfort	*Molestia*	Pain	*Incomodidad*
			Bother	
			Inconvenience	
EMPOWER screen	Becoming addicted	*Volverse adicto*	Becoming an addict	*Volverse adicto a los medicamentos*
			Becoming a drug addict	
	Being perceived as a drug-seeker	*Ser visto como un drogadicto*	Being perceived as a drug addict	*Ser percibido como un buscador de drogas*

#### Quantitative results

Over three quarters (*n* = 29, 76.3%) of respondents indicated they had received the EMPOWER materials. Among those who had received materials (*n* = 29), a majority (58.6%) indicated that they, or another staff member, had used the materials with patients or families. Respondents estimated that ∼212 hospice families had been given EMPOWER materials, nearly a third of whom (62.3%) had received Spanish versions. In terms of usefulness, using the single item rating (0 = *not useful* to 10 = *very useful*), respondents gave the English version of the EMPOWER materials an average rating of 7.6 (SD = 1.4, range = 6–10) and the Spanish version an average rating of 8.4 (SD = 1.4, range = 6–10). A majority (62.1%) of respondents indicated they would likely use EMPOWER materials in the future.

#### Qualitative results

Qualitative themes and exemplary quotes (including those referenced below) are displayed in Table 3. Among those who received the EMPOWER materials, the survey asked what was useful, if anything, about the materials. Respondents generally indicated the materials served as a good communication tool for addressing common myths and misperceptions about pain management. One participant described the perceived benefit of improving knowledge about hospice among Spanish-speaking patients (see Quote A.1.).

**Table 3.** Qualitative Themes and Exemplary Quotes by Prompt

Themes	Exemplary quotes
EMPOWER is useful because it…	
Improves knowledge of hospice	Quote A.1: *Our Spanish-speaking patients have very limited health literacy regarding hospice care in our agencies. It helps inform them of aspects of hospice. The English-speaking patients are more familiar with hospice and pain management, so less useful for them.*
Addresses opioid/morphine concerns	Quote A.2: *After discussing these eight common concerns on here… It's so to the point. I really like it. Usually, when I go out there and educate on our carepacks. Especially with the morphine, they might say, “I don't even want that ordered.” After explaining how we will always be there to instruct and monitor. They confide in you because you're not pushing it on them.*
Is culturally applicable	Quote A.3: *I think it helped a lot—especially a lot about the discussion of addiction, and the side effects to it. With the Hispanic culture, we do have a lot of “no, I've heard you become addicted to that” or “no, I've heard about the side effects.” They're very hesitant toward medications.*
Addresses stoicism	Quote A.4: *A lot of times, families don't want to take anything for pain. I think it's more of a cultural belief—especially in males. A lot of times, they think it's weakness. They want to just stick it out because there's this fear. We're so used to being brought up like “if there's pain, just stick it out.”*
Reinforces credibility/clinical education	Quote A.5.i: *If we give them a booklet or something, it helps with our credibility and it brings the family more comfort, like “they're not lying to me” and it brings family comfort, too. They can go back and refer to it, too.* Quote A.5.ii: Once we show them and explain to them, they're like “OK.” They have a better understanding because they were able to read it. They took their time and were able to reread it. They understood that it's not a bad thing to take something for pain management.
Challenges to providing EOL care to Spanish-speaking patients/families	
Clinical staff fluency	Quote B.1.i: *I don't know so much if it's the language so much as the culture but our [the younger staff's] Spanish isn't perfect—it's more the older clinicians. It's enough to get by, but it's Tex-Mex. They're more comfortable with those for whom it flows well. The hardest part is explaining end-of-life and using the correct [medical] terms (*e.g., *terminal diagnosis, specific body systems).* Quote B.1.ii: Our medical director speaks Spanish fluently. This has been a big plus in good pain management.
Spanish materials are needed on disease processes/prognosis	Quote B.2.i: *What would be most helpful would be* [written materials] *that explain medications and the ones that explain diseases process in Spanish.* Quote B2.ii: The whole death and dying process sometimes. They don't understand that they are getting closer to death. That the body is not going to need so much nutrition. The Hispanic culture—we celebrate death through Día de los Muertos—but they don't accept death. They don't do DNR [Do Not attempt Resuscitation orders]. They don't understand DNRs. They just don't understand that the way they were last week—up and walking around and drinking and eating three meals—this week, they may only eat one meal. They don't understand how change. There is a disconnect between Día de los Muertos, but, yet, we shun talking about what we want done—we don't talk about it or what we want done. It's OK when it's an abstraction but becomes fraught when it becomes real.

Another participant further acknowledged the use of the materials in addressing Spanish-speaking patients and families' concerns about potential addiction, side-effects, particularly about the fear of morphine that was emphasized by multiple respondents (see Quote A.2.). One participant also pointed out the cultural applicability of the translated EMPOWER materials to Hispanic patients (Quote A.3.). Stoicism was also described by multiple respondents as a general cultural trait that serves as a barrier to pain management (Quote A.4.). Respondents further shared that hardcopy EMPOWER materials helped reinforce the credibility of the clinical education provided by the hospice team (Quotes A.4.i. and A.4.ii).

When asked about other challenges to providing end-of-life care to Spanish-speaking patients and families, multiple respondents shared that Spanish language fluency helps to limit the challenges and using the correct terms (e.g., medical terminology) is important (Quotes B.1.i. and B.1.ii.). Similarly, respondents highlighted the value of written Spanish language materials.

In particular, several participants requested additional Spanish language education material addressing diagnosis, prognosis, and end-stage processes, and treatment options (Quotes B.2.i and B.2.ii.).

## Discussion

Using an innovative translation and low-touch dissemination approach, our study (1) successfully translated EMPOWER materials into Spanish, (2) enabled use of the materials with hospice patients and families, and (3) determined the usefulness of the materials from the perspectives of providers. All participating hospice representatives rated the EMPOWER materials—both English and Spanish versions—as either useful or very useful, and a majority indicated they were likely to use the EMPOWER materials in the future.

Coupled with past evidence,^5,6^ EMPOWER appears to be a useful, easy to use, and efficacious intervention addressing pain barriers among diverse hospice populations. For the English version, EMPOWER is poised for a large broad-based effectiveness trial. Future research, however, is needed to evaluate the efficacy of the Spanish version of EMPOWER.

The effective treatment of patient pain remains a high priority for hospice providers, and pain medications, including opioids such as morphine, remain critically important treatment options for preventing unnecessary patient suffering. However, publicity about the current U.S. national opioid epidemic^16^ and recent spike in opioid-related deaths^17^ has increased public awareness about the risks of narcotic medications. Although the risks should not be minimized, such public awareness is rarely tempered with the well-documented benefits and legacy of safe use that pain medicines have in hospice and palliative care settings.

Publicity about the opioid epidemic is likely fueling greater patient/family fears, misperceptions, and stigma about the use of pain medicines, thus, creating more barriers to pain-related communication, pain management, and medication regimen adherence within the hospice context.^18^ The evidence presented in the study herein suggests that the EMPOWER materials are a useful, feasible, and timely approach for identifying and addressing these prevalent barriers to care.

Based on our qualitative data and prior research on disparities in pain management,^18,19^ the Spanish materials appear to target culturally specific barriers to pain management, such as addiction concerns, side effects, and stoicism. This augers well for the potential effectiveness of EMPOWER among Spanish-speaking patients and families. However, if hospices lack Spanish-speaking staff, then it may be challenging for hospice families have more in-depth conversations with clinical team members about their questions and concerns regarding the many nuanced issues that surround pain management at the end of life.

Over a third of hospice agencies reported that they did not have enough Spanish-speaking staff to meet the needs of their patients, pointing to a need for more Spanish-speaking hospice clinicians. If U.S. demographic trends continue,^20^ the need for frontline clinicians who can appropriately meet the care needs of aging and linguistically diverse populations will increase. However, this study, along with several others,^21,22^ suggests a lack of preparedness in hospice to meet this challenge. Some of this may be because contexts such as hospice are constrained by a resource-limited environment^23^ and, therefore, may not be able to afford additional specialized bilingual personnel costs.

Several survey respondents identified a need for Spanish-language materials describing diagnosis, prognosis, and end-stage processes, and treatment options. Although several outlets for such information may already exist,^24–26^ it appears that at least some hospice providers are unaware of these resources—or perhaps the available materials are not meeting the informational needs of the population and require updates and modifications. Future research or public health efforts may be needed to ensure that the informational needs of Spanish-speaking patients and families are being met on a variety of clinically salient topics.

This study provides a critically important tool and promising evidence on the feasibility and utility of the EMPOWER approach with patients and families who speak either English or Spanish. Although we were unable to ask hospice patients and families about their perceptions of the EMPOWER materials, our respondent sample was relatively diverse, with over half identifying as non-White and over half indicating being Hispanic. Our efforts are designed to improve the quality of communication and care among Spanish-speaking hospice patients and families and—ultimately—reduce obstacles to pain management in hospice.

Results may also inform future translation efforts of EMPOWER materials into other common languages spoken in the United States, such as Chinese, Korean, Japanese, French, Tagalog, and Vietnamese. Given the unique cultural contexts of these language-centered communities, the EMPOWER protocol may require significant modification to facilitate successful implementation, particularly in settings with fewer resources or limited access to hospice.

Study results should be interpreted in consideration of certain limitations. Despite a robust sampling framework comprising all U.S. hospices with ≥25% Hispanic patient representation, participation in the follow-up survey was low and the resulting sample size small, constraining generalizability and raising concerns about non-response bias (e.g., perhaps hospices that used the EMPOWER materials were more likely to participate). Further research is needed with larger samples to replicate findings and more accurately estimate population parameters. Previous hospice researchers have observed similar challenges to engaging hospice staff members in research, including concerns about gatekeeping, high turnover, industry consolidation, and a general reluctance to participate due to limited time.^27^

Our results relied exclusively on perspectives of hospice representatives. Future research is needed to understand the experiences of hospice patients and families who receive the EMPOWER intervention. The self-reported nature of our data is subject to social desirability bias and, thus, participants may have overstated the benefits and usefulness of EMPOWER. This being stated, qualitative data from participants often provided descriptions of the underlying rationale for positive assessments of EMPOWER, lending greater credibility to the quantitative results.

Despite several limitations, this study adds critically needed evidence on a promising and pragmatic intervention to identify and overcome common barriers to pain management in hospice. Spanish-translated EMPOWER materials may be a viable tool for minimizing known disparities in pain management at the end of life among Hispanic patients and their informal caregivers. The study, although small, also provides needed empirical data on the state of Spanish language/bilingual resources in the hospice setting.

## Supplementary Material

Supplemental data

## Supplementary Material

Supplementary Appendix SA1
