# Supplementary material for: Spanish Translation and Dissemination of EMPOWER Materials to Address Barriers to Pain Management at the End of Life
Source: Palliat Med Rep. 2024 Apr 15;5(1):162–70. doi: 10.1089/pmr.2023.0090 (PMC11043617; doi:10.1089/pmr.2023.0090)
Supplement: Supplemental data [file Suppl_AppendixSA1.pdf]

1. Interviewer

[INTERNAL USE ONLY]

Interviewer initials:

[INTERNAL USE ONLY]

Please indicate the name and zip code of your hospice (so we can confirm that the survey has been completed for your organization and that no further contact from the study team is required).

Hospice name

Zip code

2. Informed Consent

This is an approximately 5-minute survey regarding resources for Spanish-speaking hospice patients and families. The informed consent document below will tell you more about the study:

Researchers at the University of Maryland and the University of South Florida are conducting an evaluation of how hospices address the needs of Spanish-speaking families to determine whether Spanish-translated EMPOWER materials are useful. This study seeks input from hospices whose

patient populations are at least 25% Hispanic, as identified by the Centers for Medicare & Medicaid Services's Provider of Services file.

Procedures: You will complete a survey in your preferred format (e.g., phone-based or self-administered) on issues related to pain management in your agency. The survey will take about 5 minutes to complete and should be completed in one sitting (versus starting and stopping) in a setting with minimal distractions.

Benefits: You will not benefit directly from your participation in this study. Hospices, however, may benefit from continued access to EMPOWER materials free of charge. Results from this study will provide insight into clinical staff's perceptions of pain management for Hispanic patients at the end of life, which may be used to improve care provision.

Risks: This study will seek information at the agency level. Thus, the risks of participation to participants are minimal. You will complete a survey that is specific to your agency, which will inform the research team of the agency for which you work. However, this information cannot be used to identify individuals. A minority of questions will inquire about general, personal information. Data from the study may be published; however, no identifying information will be used in reports or publications.

Compensation: No compensation will be offered for participation in this survey.

Participation: You are not required to participate in this study. The decision to participate in this study is completely voluntary and you can stop at any time.

If you have questions, concerns, or complaints, please contact John Cagle, PhD, MSW, who is Principal Investigator on the study (JCAGLE@ssw.umaryland.edu; 410-706-6106). For concerns about your rights as a participant, please contact the University of Maryland, Baltimore Human Research Protections Office (hrpo@umaryland.edu; 410-706-5037).

By clicking the arrow, you are providing informed consent to participate.

3. Participant Professional Background

This first section will inquire about your professional background.

What is your job role?

[ ]

Is your position clinical, administrative, or both?

- ○ Clinical
- ○ Administrative
- ○ Both

Hospice is interdisciplinary; so, what is your professional background? [Check all that apply.]

- ☐ Medicine
- ☐ Nursing
- ☐ Nursing aide
- ☐ Social work
- ☐ Pharmacy
- ☐ Chaplain or spiritual services
- ☐ Volunteer
- ☐ Other: Self-describe ________

4. Bilingual Staff at Agency

This next section will inquire about bilingual staff at your agency. When we say "bilingual," we refer to someone who is fluent in both English and Spanish.

Does your agency employ at least one full-time hospice team member (i.e., a provider of direct patient care) who is bilingual (English–Spanish)?

- ○ No
- ○ Yes

Approximately how many bilingual (English–Spanish) hospice team members are employed at your agency?

- ○ 0
- ○ 1
- ○ 2
- ○ 3–5
- ○ 6+
- ○ Don't know

Which of the following patient care disciplines have bilingual (English–Spanish) speakers at your agency?

	No	Yes	Don't know
Medicine	○	○	○
Nursing	○	○	○
Nursing aide	○	○	○
Social work	○	○	○
Pharmacy	○	○	○
Chaplain or spiritual services	○	○	○

	No	Yes	Don't know
Volunteer	○	○	○

Would you say that your hospice employs enough Spanish-speaking clinical staff to meet the needs of your patients?

- ○ No
- ○ Yes

5. Interpretation Services

This next section will inquire about interpretation services at your hospice.

To the best of your knowledge, does your agency use any English–Spanish interpretation services outside of those provided by your agency's clinical staff?

- ○ No
- ○ Yes

Do you think that these outside interpretation services are needed at your agency?

- ○ No
- ○ Yes

Please explain why you do or do not think these outside interpretation services are needed at your agency.

Please briefly describe the interpretation services provided at your agency.

6. Experience With EMPOWER Materials

This next section will inquire about the EMPOWER materials to help address barriers to pain management that we mailed to your hospice. We sent some hard-copy screening forms and brochures.

To be clear, the following questions do not refer to the follow-up postcard that we also mailed.

Did your agency receive the EMPOWER screening form and brochures in the mail or via email?

- No
- Yes

Have you or anyone at your agency used any of the EMPOWER materials with patients or families?

- No
- Yes

- ○ Don't know

Please describe why these materials were not used. There are no wrong answers.

Please indicate the version(s) of the materials you used.

- ○ English
- ○ Spanish
- ○ Both

Approximately how many households received each version of these materials? (Enter numeric digits only.) [If not applicable, please enter 0.]

English

Spanish

Please rate the usefulness of the EMPOWER materials from 0 (*not useful*) to 10 (*very useful*)? [If not applicable, please leave blank.]

	0	1	2	3	4	5	6	7	8	9	10
English	○	○	○	○	○	○	○	○	○	○	○
Spanish	○	○	○	○	○	○	○	○	○	○	○

Please briefly describe the ways in which these EMPOWER materials were useful in patient care.

7. Future Use of EMPOWER Materials

This next section will inquire about any future use of the EMPOWER materials.

Are you likely to use the EMPOWER materials in the future?

- No
- Yes

Please briefly explain your answer above. There are no wrong answers.

Do you have any recommendations for improving the EMPOWER materials?

8. General Challenges in Serving Patients Who Only Speak Spanish

Without divulging any patient identifiers, can you share about challenges/successes in addressing pain management with patients who only

speak Spanish?

What are the other biggest challenges when caring for patients who only speak Spanish?

9. Demographics

This final section will inquire about your demographic information.

Please indicate your age in years. [Enter numeric digits only.]

[INTERVIEWER NOTE: IF UNKNOWN] Please indicate your gender.

- Male
- Female
- Other gender identity

Please indicate your ethnicity.

- Hispanic or Latino
- Not Hispanic or Latino

Please indicate your race. [Check all that apply.]

- ☐ American Indian or Alaska Native
- ☐ Asian
- ☐ Black or African American
- ☐ White
- ☐ Native Hawai‘ian or Other Pacific Islander
- ☐ Other: self-describe ______

10. Todd's Questions

Thank you for your input regarding the EMPOWER materials.

There are two additional, optional questions that are unrelated to EMPOWER but instead pertain to medical aid in dying. May we ask you those questions?

- ○ No
- ○ Yes

In recent years, there has been a progression of legalization of medical aid in dying (aka "physician-assisted suicide" and "death with dignity") across states in the U.S.

How has your agency prepared, if at all, to accommodate requests for medical aid in dying?

Given the high proportion of Hispanic patients served by your hospice, what cultural considerations, if any, need to be made in order to accommodate requests for medical aid in dying?

11. Thank You

Thank you for participating in this survey! Your responses have been documented.

If you have questions, concerns, or complaints, please contact John Cagle, PhD, MSW, who is Principal Investigator on the study (JCAGLE@ssw.umaryland.edu; 410-706-6106). For concerns about your rights as a participant, please contact the University of Maryland, Baltimore Human Research Protections Office (hrpo@umaryland.edu; 410-706-5037).

Powered by Qualtrics
